# Complete Genome Sequence of Lactobacillus plantarum Strain B21, a Bacteriocin-Producing Strain Isolated from Vietnamese Fermented Sausage Nem Chua

**DOI:** 10.1128/genomeA.00055-15

**Published:** 2015-04-02

**Authors:** Aida Golneshin, Eric Adetutu, Andrew S. Ball, Bee K. May, Thi Thu Hao Van, Andrew T. Smith

**Affiliations:** School of Applied Sciences, RMIT University, Melbourne, Victoria, Australia

## Abstract

Lactobacillus plantarum strain B21 was isolated from Vietnamese sausage (nem chua) and demonstrated broad antimicrobial activity due to the production of bacteriocins. Here, we report the complete genome sequence of this strain (3,284,260 bp).

## GENOME ANNOUNCEMENT

Current interests in lactic acid bacteria (LAB), especially Lactobacillus plantarum, have increased because of their application as starter cultures in food fermentation (1, 2). LAB produce a variety of compounds with antimicrobial activity, including acids, hydrogen peroxide, and bacteriocins (3). The antimicrobial activity of bacteriocins provide an advantage for the growth of the producer strain, creating a selective microenvironment (4). Probiotic strains with antimicrobial activity have unique advantages as starter cultures in food applications. The L. plantarum strain B21 was isolated from the Vietnamese fermented sausage nem chua (5) and showed activity against a wide range of Gram-positive bacteria due to the production of bacteriocins. To date, there are only six L. plantarum strains that have been completely sequenced and annotated. Here, we report the gapless chromosome sequence of L. plantarum strain B21.

The B21 genome was sequenced using the Illumina HiSeq 2000 sequencing platform (BGI, China). A total of 527 Mb of data was produced with next-generation Illumina paired-end sequencing technology. A genome library containing different fragment length insertions (500 bp and 6 kb) was constructed, and 6,354,788 paired-end 90-bp reads were generated with 164-fold coverage. The *de novo* assembly was performed using SOAPdenovo (6, 7) and consisted of 29 contigs and 20 scaffolds. Contigs were further assembled using multiple assembly software. Finally, the SOAPdenovo software was used in combination with a PCR approach to close the gaps and correct the misassemblies. This resulted in an optimized assembly consisting of one final super scaffold and one contig. The Glimmer version 3.0 program was used to conduct gene prediction and the obtained gene sequences were compared using the NCBI Prokaryotic Genome Annotation Pipeline, for functional gene annotation. The rRNAmmer, tRNAscan, and Rfam software were used to predict rRNA, tRNA, and sRNA, respectively (8–10).

The complete genome sequence of L. plantarum B21 showed one circular chromosome of 3,284,260 bp with a GC content of 44.47%. The chromosome contains 3,117 genes (including 2,930 coding sequences, 51 pseudogenes, and 18 frameshifted genes) with a total length of 2,766,912 bp, which made up 84.3% of the genome. The average gene length was 887 bp, and the GC content in the gene region was 45.5%. The number of rRNA, tRNA, and sRNA genes was 17, 65, and 2, respectively. There is ambiguous evidence for the presence of a number of natural plasmids in this strain, which are currently under further investigation.

Comparison of the B21 genome with the L. plantarum WCFS1 reference strain (Genbank accession number AL935263.2) revealed that 2,668 out of 3,117 genes (80.17%) of B21 aligned well with those of WCFS1 with a mean identity of 99%. There were 449 unique genes in the B21 genome that did not align to WCFS1.

The significant number of transpositions relative to its nearest neighbor WCFS1, confirm the well-known plasticity of the LAB genome and this Vietnamese isolate.

### Nucleotide sequence accession number.

The complete genome sequence of L. plantarum B21 has been deposited in NCBI GenBank under the accession number CP010528.
